# Perceptions of Pakistani medical students about drugs and alcohol: a questionnaire-based survey

**DOI:** 10.1186/1747-597X-1-31

**Published:** 2006-10-25

**Authors:** Majid Shafiq, Zaman Shah, Ayesha Saleem, Maham T Siddiqi, Kashif S Shaikh, Farah F Salahuddin, Rizwan Siwani, Haider Naqvi

**Affiliations:** 1 MBBS Class of 2006; Medical College, Aga Khan University, Stadium Road Karachi 74800, Pakistan; 2 Department of Psychiatry, Aga Khan University, Stadium Road Karachi 74800, Pakistan

## Abstract

**Background:**

Drug abuse is hazardous and known to be prevalent among young adults, warranting efforts to increase awareness about harmful effects and to change attitudes. This study was conducted to assess the perceptions of a group of medical students from Pakistan, a predominantly Muslim country, regarding four drugs namely heroin, charas, benzodiazepines and alcohol.

**Results:**

In total, 174 self-reported questionnaires were received (87% response rate). The most commonly cited reasons for why some students take these drugs were peer pressure (96%), academic stress (90%) and curiosity (88%). The most commonly cited justifiable reason was to go to sleep (34%). According to 77%, living in the college male hostel predisposed one to using these drugs. Sixty percent of students said that the drugs did not improve exam performance, while 54% said they alleviated stress. Seventy-eight percent said they did not intend to ever take drugs in the future. Females and day-scholars were more willing to discourage a friend who took drugs. Morality (78%), religion (76%) and harmful effects of drugs (57%) were the most common deterrents against drug intake. Five suggestions to decrease drug abuse included better counseling facilities (78%) and more recreational facilities (60%).

**Conclusion:**

Efforts need to be made to increase student awareness regarding effects and side effects of drugs. Our findings suggest that educating students about the adverse effects as well as the moral and religious implications of drug abuse is more likely to have a positive impact than increased policing. Proper student-counseling facilities and healthier avenues for recreation are also required.

## Background

Substance abuse is defined as a maladaptive pattern of substance use leading to clinically significant impairment or distress, wherein the person may also suffer from tolerance and withdrawal [1].

Substance abuse is a common problem worldwide. Pakistan, a South Asian developing country with a population of 150 million, is no exception. Ninety seven percent of Pakistan's population is Muslim, with highly conservative customs and traditions governing the lives of many. All substances of abuse are publicly despised, but none more than alcohol, which finds a direct reference in the Koran. This absolute taboo is in stark contrast to the acceptability that alcohol finds in the Western society. Nonetheless, alcohol is widely consumed by various sections of the society, most notably by the very affluent and the impoverished.

Cultivation of poppy has been carried out in the northern part of Pakistan for a long time. During the British rule, opium was sold in licensed shops throughout the Indo-Pak subcontinent. At the time of independence in 1947, there were approximately 100,000 regular and registered opium users in Pakistan [2].

In 1979, the Islamic Revolution in Iran, the Soviet invasion of Afghanistan and the enforcement of Hadd Ordinance in Pakistan directly or indirectly affected the geopolitical situation in the country. [3] The ordinance outlawed cultivation, production, distribution and sale of all substances of abuse including opium, charas and alcohol. If anything, however, the same period marked a substantial increase in the consumption of such substances in Pakistan.

The events in Iran and Afghanistan drove nearly 5 million refugees into Pakistan. A number of them were involved in cultivation, production and smuggling of drugs, including heroin. Heroin was virtually not known in Pakistan prior to 1979. On the other hand, the Hadd Ordinance drove the drug trade underground and led to the emergence of drug mafia. Existence of long established routes in Pakistan for trade to and from Afghanistan provided convenient and organized channels for drug smuggling.

According to the 5th and last national survey (National Survey on Drug Abuse – N.S.D.A) conducted in 1993 by the Pakistan Narcotic Control Board, there were nearly 3 million drug dependents in Pakistan with 51 % of them being heroin dependents. [2]. This represents nearly a three fold increase in the total number of dependents and 30 fold increase in the number of heroin dependents when compared to the findings in the 1st N.S.D.A report of 1982.

Survey data was based on community based samples using robust case ascertainment methods. Although the government of Pakistan had envisaged conducting these national surveys every five years, no further survey have been conducted since 1993. The Pakistan Narcotic Control Board (PNCB) was disbanded in the mid 90s. Presently an Anti Narcotic Force (A.N.F) operates in order to serve as narcotic control and enforcement agency with no mandate to carry out steps toward drug abuse prevention or long term rehabilitation.

Among the drugs of abuse, heroin is the most commonly abused drug followed by hashish, charas, (the latter two derived from resinous exudates of the flowering tops of female Cannabis sativa plant) bhang, opium, alcohol and psychotropic drugs [2].

The medical personnel are vulnerable to substance abuse and dependence due to ready access to substances of abuse. Many studies have estimated this prevalence among students of the medical sciences. According to one study, there is a higher occurrence of misuse of alcohol, tranquillizers and psychedelics among medical students, and dependence rates are 5% for medical students and 3% for doctors [4]. A study conducted among undergraduate medical students in two medical colleges of Calcutta indicated that during 1993, the point prevalence values of total and current drug abusers were 48.9% and 27.9% respectively among the respondent student population [5]. Turkey, a predominantly Muslim country (as is Pakistan), has reported point prevalence figures of only 4% for the use of illicit drugs (cannabis, ecstasy, cocaine) among medical students. However, 46.1% of the students consumed alcohol, among which 7.4% had risky alcohol use [6].

It is plausible that the medical personnel's knowledge and understanding of drugs of abuse is overestimated. Clearly, knowledge about the desirable and undesirable effects of a drug may significantly alter the drug's usage. We therefore aim to assess the knowledge and attitude of undergraduate students of a private medical college regarding drugs, including alcohol. To our knowledge no such study has previously been published from Pakistan.

## Methods

This was a cross-sectional study conducted among the undergraduate students of a private medical university in Karachi. Most of the university's students hail from affluent families. Like all institutions in Pakistan, it offers a five-year course in "bachelor of medicine and bachelor of surgery" (M.B., B.S.). Students typically get enrolled into this course immediately after completing high school, i.e. at around 18 years of age. In order to become a registered medical practitioner, the M.B., B.S. graduate has to do one year of internship, which comprises of six months' training in medicine and another six in surgery.

The study was conducted in compliance with 'Ethical principles for medical research involving human subjects' of Helsinki Declaration. Study protocol was discussed among the students and facilitating faculty for possible ethical concerns. All possible measures were taken to ensure the confidentiality of all participants. Verbal informed consent was obtained from the subjects.

Two hundred subjects were selected through convenience sampling and a standard, pre-tested questionnaire was administered in English language. Participants were allowed to return the filled questionnaire forms to any one of the persons designated by investigators within 15 days.

The questionnaire was formulated on the basis of thorough review of literature, after detailed discussions and peer-review among investigators and facilitating faculty. The preliminary questionnaire was pre-tested on 25 students and modified to address the identified deficiencies.

The first part of the questionnaire sought information related to demographics of the participants. Perceptions of students regarding four common drugs of abuse namely alcohol, charas, heroin and benzodiazepines (BDZs) were assessed in the second part of the questionnaire. This included their beliefs regarding possible beneficial (e.g. stress alleviation) or harmful effects of these drugs, factors predisposing to initiation and continuation of drugs, factors deemed justifiable to use drugs, and their possible reaction to a colleague taking drugs. They were also asked whether they intended to ever take drugs in the future.

In order to exclude interviewer bias, the terms "misuse" and "abuse" were avoided in the questionnaire and the term "use" or "take" was employed instead.

Respondents were not asked about their own drug and alcohol practices. This was done in order to avoid discouraging students from participating, since drugs and alcohol are a taboo subject in our society as mentioned earlier.

Data was entered and analyzed in Statistical Package for Social Sciences 13.0 (SPSS 13.0). Descriptive statistics of socio-demographic information and perceptions were determined. Chi square test was used to examine putative associations between perceptions and demographic variables. For all purposes, a p-value of <0.05 was considered as the criteria of significance.

## Results

Out of the 200 questionnaires distributed, 174 filled questionnaires were received (87% response rate). There were 96 males and 78 females. The ages ranged from 18 to 25 years, with a mean of 21.3 years. Table 1 illustrates the other demographic characteristics.

**Table 1:** Demographic characteristics of the study subjects

**VARIABLE**		**SUBJECTS (%)**
SEX	MALE	96 (52)
	FEMALE	78 (48)
YEAR OF UNDERGRADUATE MEDICAL COURSE	1^ST^	37 (20)
	2^ND^	30 (16)
	3^RD^	38 (21)
	4^TH^	54 (29)
	5^TH^	25 (14)
RESIDENCE	HOSTELLITE	130 (71)
	DAY-SCHOLAR	54 (29)

**Figure 1:**
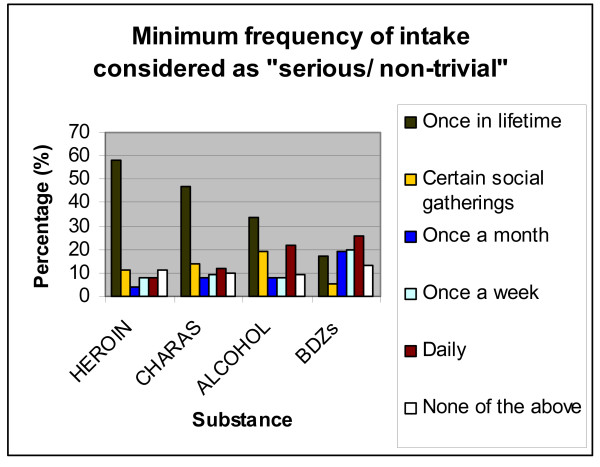
Minimum frequency of intake considered by the respondents as "serious/non-trivial".

Figure 1 illustrates the frequency of each of the substances under study perceived by the students to be serious/non trivial. For alcohol, even once-in-a-lifetime use was considered serious by 34%, while another 22% said that daily use (or more) was non-trivial. Forty-seven percent said that once-in-a-lifetime use of charas was non-trivial, while 14% cited intake at only social gatherings to be the minimum serious frequency. Once-in-a-lifetime use of heroin was considered serious by 58%, and use at only social gatherings by another 11%. For BDZs, 26% said that a daily use would be the minimum serious frequency, while another 20% cited once-a-week as that rate.

Table 2 illustrates the most common factors perceived as predisposing students to taking drugs. Consumption of drugs by friends and consumption by family members were among the most common predisposing factors identified (90% and 74%, respectively), as was tobacco smoking (76%).

**Table 2:** Factors perceived by respondents as predisposing to drug usage among students

**PREDISPOSING FACTOR**	**FREQUENCY (%)**
FRIENDS USING DRUGS	166 (90)
TOBACCO SMOKING	139 (76)
FAMILY USING DRUGS	136 (74)
CERTAIN SOCIAL GATHERINGS	128 (70)
LIVING AWAY FROM HOME	114 (62)
MALE GENDER	98 (53)
LOW SELF-ESTEEM	93 (51)
INADEQUATE RECREATIONAL FACILITIES	91 (50)

When asked to identify the reasons for students taking up drugs, respondents cited peer pressure (96%), academic stress (90%), curiosity/for experimentation and "to get high" (88% each) as the leading ones. To sleep (34%), academic stress (20%) and curiosity/for experimentation (20%) were the leading reasons deemed justifiable by the respondents.

Top reasons why certain students do not take drugs included moral unacceptability (78%) and religion (76%). "Harmful effects of drugs" was cited as a reason by 57% of respondents, while 38% cited "fear of being caught" as one reason.

Ten percent of the respondents said that drugs improved performance in exams, 60% said drugs did not, while 30% said they did not know. According to 54%, drugs helped alleviate stress, 18% said drugs did not, while 28% said they did not know. For each of these questions, a higher proportion of respondents belonging to the senior or 'clinical' years (4^th ^and 5^th^) replied in the negative (chi-square = 12.3, df = 2, p = 0.01 and chi-square = 10.2, df = 2, p = 0.01 respectively).

Seventy-seven percent said that living in the college male hostel predisposed one to using drugs, the top reasons being greater peer pressure (65%), easier access to drugs (59%), lack of parental influence (58%) and greater exposure to stresses (44%). According to 22%, living in the female hostel predisposed one to using drugs.

Seventy-eight percent of the respondents said they did not intend to ever take drugs in the future, 10% said they did and nine percent said they did not know. Seventy-one percent of males and 89% of females said they would discourage a colleague using drugs; gender was associated with reaction to a colleague's use of drugs (chi-square = 42.3, df = 1, p = 0.01). Day-scholars were more willing than hostellers to discourage their colleagues (chi-square = 24.8, df = 1, p = 0.01).

Respondents were asked to identify adverse outcomes associated with intake of the afore-mentioned drugs. Addiction (92%), threat to own or others' lives (80%), socially inappropriate behavior (72%) and diminished academic performance (67%) were the leading problems recognized. When asked to identify adverse outcomes specifically associated with alcoholism, 95% cited chronic liver disease. This was followed by depression (65%), neurological illness (60%) and reduced life expectancy (58%). Those who identified at least three adverse outcomes were more likely to consider a less than daily use of alcohol as serious/not-trivial (chi-square = 3.9, df = 1, p = 0.05).

Lastly, respondents were asked to make suggestions on how to reduce the problem of drug use on campuses. The most common responses were availability of student counseling facilities (78%), more recreational facilities on campus (60%), more frequent hostel checking by the security staff (51%) and drug rehabilitation programs (48%).

## Discussion

In our study, different levels of acceptability were observed for different substances (figure 1). Respondents were more likely to be comfortable with an occasional intake of alcohol as compared to charas or heroin. BDZs had an even higher acceptance among the participants. We feel that the low threshold for use of BDZs reflects its widespread acceptability as a relatively benign sleeping pill, which is supported by the observation that "to get to sleep" was the most commonly cited justifiable reason to take drugs. The relative acceptability of alcohol over charas or heroin is evidently due to a lack of knowledge about its harmful effects, since respondents who identified at least three adverse outcomes were more likely to consider a less than daily use of alcohol as serious. In the same vein, "harmful effects of drugs" was a commonly cited reason for certain students not taking up drugs.

Many of the common factors predisposing to drug misuse perceived by our subjects have already been established through Western studies on college students. These include peer involvement, living away from home, poor self-esteem and male gender [1,2,5,7]. According to a study conducted among Thai adolescents, male gender was a risk factor for every untoward drug-related behavior. Poor self-esteem was also a risk factor, while socio-environmental factors included being in a gang and loneliness [8].

The most common reasons for students misusing drugs identified by our subjects were peer pressure (96%), academic stress (90%), curiosity/for experimentation (89%) and "to get high" (88%). According to a study carried out on a Norwegian population, curiosity and peer pressure were the main reasons for starting drugs. Family conflicts, school and mental problems were each reported by about 40% of the subjects [7]. Of note, not only was academic stress the second most commonly cited reason among our respondents, it was also the second most common reason deemed to justify drug intake.

Tobacco smoking was identified as a predisposing factor by 76% of our respondents. According to a study carried out in the United States, those who had smoked cigarettes were more likely to consume cocaine (OR = 7.5), heroin (OR = 16.0), crack (OR = 13.9) and marijuana (OR = 7.3). These associations were consistent across age-strata and remained after adjusting for race and gender [9]. This and other studies suggest that cigarette smoking may be a gateway drug to illegal drugs as well as to alcohol, although that is not conclusively proven yet [10,11].

According to seventy-seven percent of our respondents, living in the campus' male hostel predisposed one to misusing drugs. According to a study carried out in neighboring India, too, more hostellers were found to be drug consumers than non-hostellers [4]. When our respondents were asked to state the reasons, they cited greater peer pressure (65%), easier access to drugs (59%), lack of parental influence (58%) and greater exposure to stresses (44%), among others. Notably, half of all respondents cited "inadequate recreational facilities" as a predisposing factor to drug misuse (Table 2); it is possible that this factor may have played an important role in the hostellers' inclination toward drugs. Students need to be shown that there are better, safer ways to enjoyment than resorting to drugs.

In conformity with our subjects' views, religiosity has been seen to be associated with lower frequencies and quantities of alcohol intake [4]. It is also conceivable that a greater knowledge of harmful effects of drugs would lead to decreased consumption of the same, since our subjects were clearly deficient in such knowledge. Moreover, over half of all respondents cited "harmful effects of drugs" as a reason why certain students do not take up drugs.

As already mentioned, a commonly identified predisposing factor was living in the campus male hostel. Steps undertaken by Western institutions to curb drug consumption in hostels include cutting access to drugs, greater checking and swifter punishments [12]. However, our study suggests that students possess a mature sense of censorship that is responsive to moral and medical reasoning: 78% cited "moral unacceptability" as a reason for students not taking up drugs and 76% cited "religion". Fifty-seven percent cited "harmful effects of drugs" as a deterrent while only 38% cited "fear of being caught" as a reason for abstinence. This suggests that our students are more willing to pay attention to moral, religious and medical reasoning than to increased policing. Besides, cutting students' access to drugs would be much easier said than done.

Academic stress was identified as a very important factor in drug misuse. This illustrates the pressure felt by the students to excel in their studies, which could result from overly competitive environments or from very high expectations placed by teachers, family and/or friends. A conscious effort needs to be made in alleviating this pressure as much as possible without unduly decreasing the need felt by the students to study.

A major limitation of our study was that it was carried out on students from a single medical college. Moreover, we chose a private medical college, which has a higher tuition fee as compared to public medical colleges across the country. Therefore, students of this college are likely to belong to more affluent and more educated families on average than students of a typical public medical college in Pakistan, thereby introducing a selection bias.

## Conclusion

We conclude that undergraduate medical students are inadequately aware of the effects and side effects of common drugs of abuse. The relative acceptance of BDZs is a point of particular concern. Much needs to be done in order to increase awareness and change attitudes towards this hazard.

Our findings suggest that educating students about the dangers of drug intake as well as its moral and religious implications is likely to be more beneficial than increased policing. Integration of addiction medicine into the undergraduate medical curriculum might be of value for the professional development of the aspiring doctors. In this regard, guidance can be sought from Western recommendations such as the one made by 2004's Leadership Conference on Medical Education in Substance Abuse [13]. Efforts need to be made to make students understand that experimental use of drugs may lead to abuse and dependence later on. Importantly, efforts need to be begun at around high-school level, since that is a common age for developing such habits [14].

Students also need to be shown healthier coping alternatives and avenues for recreation.

For those who have trouble quitting drugs, a counseling and support system needs to be made readily available. Such a system should be independent in the sense that the counselors should not be academic evaluators of these students. Students need to be assured that they will be helped rather than victimized for admitting their problems. An assurance such as that along with easy accessibility and absolute confidentiality are required in order to win the trust of needy students.

## Declaration of competing interests

The author(s) declare that they have no competing interests.

## Authors' contributions

MS participated in designing the study, data gathering and literature search. He also carried out statistical analysis and drafted the manuscript. ZS and AS each participated in study designing, data gathering, literature search and manuscript writing. MTS conceived of the study and participated in its designing as well as in data gathering. KSS participated in study designing and data gathering and helped draft the manuscript. FFS participated in study designing, data gathering and literature search. RS was involved in study designing and data gathering. HN participated in study designing and coordination and helped draft the manuscript. All authors read and approved the final manuscript.
